# Inhibiting myostatin signaling partially mitigates structural and functional adaptations to hindlimb suspension in mice

**DOI:** 10.1038/s41526-022-00233-4

**Published:** 2023-01-16

**Authors:** Andrea M. Hanson, Mary H. Young, Brooke C. Harrison, Xiaolan Zhou, H. Q. Han, Louis S. Stodieck, Virginia L. Ferguson

**Affiliations:** 1 Aerospace Engineering Sciences, BioServe Space Technologies, University of Colorado, Boulder, CO USA; 2 Department of Molecular Cellular and Developmental Biology, University of Colorado, Boulder, CO USA; 3 Amgen Inc., Thousand Oaks, CA USA; 4 Department of Mechanical Engineering, University of Colorado, Boulder, CO USA; 5 BioFrontiers Institute, University of Colorado, Boulder, CO USA; 6 Present Address: AliveGen USA Inc., Thousand Oaks, CA USA

**Keywords:** Physiology, Biophysics, Anatomy

## Abstract

Novel treatments for muscle wasting are of significant value to patients with disease states that result in muscle weakness, injury recovery after immobilization and bed rest, and for astronauts participating in long-duration spaceflight. We utilized an anti-myostatin peptibody to evaluate how myostatin signaling contributes to muscle loss in hindlimb suspension. Male C57BL/6 mice were left non-suspended (NS) or were hindlimb suspended (HS) for *14 days* and treated with a placebo vehicle (P) or anti-myostatin peptibody (D). Hindlimb suspension (HS-P) resulted in rapid and significantly decreased body mass (−5.6% by *day 13*) with hindlimb skeletal muscle mass losses between −11.2% and −22.5% and treatment with myostatin inhibitor (HS-D) partially attenuated these losses. Myostatin inhibition increased hindlimb strength with no effect on soleus tetanic strength. Soleus mass and fiber CSA were reduced with suspension and did not increase with myostatin inhibition. In contrast, the gastrocnemius showed histological evidence of wasting with suspension that was partially mitigated with myostatin inhibition. While expression of genes related to protein degradation (*Atrogin-1* and *Murf-1*) in the tibialis anterior increased with suspension, these atrogenes were not significantly reduced by myostatin inhibition despite a modest activation of the Akt/mTOR pathway. Taken together, these findings suggest that myostatin is important in hindlimb suspension but also motivates the study of other factors that contribute to disuse muscle wasting. Myostatin inhibition benefitted skeletal muscle size and function, which suggests therapeutic potential for both spaceflight and terrestrial applications.

## Introduction

Exposure of astronauts to reduced gravity during spaceflight disrupts homeostasis in musculoskeletal tissues leading to atrophy and loss of strength. In both humans and rodents, skeletal muscle atrophy during spaceflight predominantly occurs in weight-bearing hindlimb muscles including those responsible for plantar and dorsiflexion, knee and hip extension, and postural control^1–3^. For astronauts on board the International Space Station (ISS), even high-volume resistance exercise does not fully prevent muscle atrophy and weakness^2,4^ and might become infeasible if astronaut health were compromised. Pharmaceutical therapies that promote or preserve muscle mass and strength, alone or in combination with exercise or dietary interventions, are essential to maintain astronaut fitness during long-duration spaceflight missions. An improved understanding of mechanisms that underlie spaceflight-induced muscle loss is thus needed.

Hindlimb suspension in mice and rats is an accepted method of simulating disuse from spaceflight^5^. Atrophy of hindlimb muscles results from rapid adaptation to unloading and includes decreased skeletal muscle mass and strength, and fiber type alterations^6,7^. In hindlimb-suspended mice, protein degradation follows upregulation of MAFbx/Atrogin-1 and MuRF-1 atrogenes, which are regulated by the Forkhead box O (FOXO) family of transcription factors, while Akt levels and downstream protein synthesis are decreased^8–10^. As in human spaceflight, the effects are most pronounced in the hindlimb plantarflex and postural muscles^9,11,12^. Hindlimb suspension thus enables the evaluation of physiological effects of disuse, associated molecular mechanisms, and assessment of therapeutics.

Yet hindlimb suspension is a complex model that mimics certain aspects of spaceflight where muscle contraction and movement can still occur^7^. Hindlimb suspension also creates a head-down tilt condition, causing a cephalic fluid shift similar to that which occurs in astronauts. Stress is increased through activation of the hypothalamic-pituitary-adrenal (HPA) axis leading to elevated glucocorticoids, primarily corticosterone^13^. Suspended mice lose up to 10% of their body weight in the first two days of suspension^9^. Weight loss at early timepoints in suspension is likely related to stress-induced reductions in food and possibly water consumption^14^; we observed where lower food and water consumption in growing (~10–11 week old) male C57BL/6 mice in the first two days of hindlimb suspension, after which food intake increased but remained below that of unsuspended controls^9^. Fasting causes muscle atrophy without increases in the glucocorticoid corticosterone^14,15^. Glucocorticoid production is high in the first few days during suspension and remains modestly elevated beyond one week^16,17^. Muscle atrophy is known to occur from disuse^18^, from elevated glucocorticoids^19^, and from short-term fasting^14^. However, the precise contribution and time course of each condition to hindlimb-suspension-induced muscle atrophy are unclear.

Myostatin, or growth and differentiation factor-8 (GDF-8), is a member of the TGF-β super-family and acts as a negative regulator of skeletal muscle^20^. Natural mutations of the myostatin gene result in muscle hypertrophy^21–23^, while overexpression causes muscle atrophy^24^. Notably, myostatin expression is increased in a number of pathophysiological states that cause muscle wasting, including disuse^25,26^, and is involved in key signaling pathways that mediate skeletal muscle turnover^20,27–29^. Myostatin negatively regulates protein synthesis via the Akt pathway by inhibiting Akt/p70s6k activation^30^ and signaling^31^. Myostatin increases protein degradation through activation of the ubiquitin–proteasome system^10^ and is mediated through increased expression of the ubiquitin e3 ligases Atrogin-1 and MuRF-1^31–33^. Therapies targeting the myostatin pathway thus hold promise to mitigate muscle atrophy that accompanies disease and disuse^34,35^.

Myostatin protein expression is increased with disuse in humans following prolonged bed rest^26,36^, unilateral lower limb suspension^37^, and hip replacement^25^. In rats and mice, myostatin increased with hindlimb unloading^12,38,39^ and microgravity^40–42^, although a spaceflight-induced myostatin increase is not a universal finding^43^. With hindlimb suspension, myostatin expression increases to peak levels in the first few days^12^ and remains partially elevated^38,39^. Mstn(-/-) mice undergo significant skeletal muscle hyperplasia and hypertrophy during development^20^, and yet these knockout mice are more prone to atrophy from hindlimb suspension than controls^44^. While this latter finding may indicate that myostatin is not critical for disuse muscle atrophy, these results must be contextualized by the lifelong absence of functional myostatin. Pharmacological inhibitors of myostatin partially mitigated muscle atrophy with hindlimb suspension^45^ or casting^46^. Latres et al.^45^ demonstrated that myostatin inhibition normalized the expression of MuRF-1 and Atrogin-1 expression, and modestly increased muscle weights, after 14 days of casting; however, muscle mass and functional performance were not increased at 21 days. Longer-term inhibition of myostatin may thus be less effective at preventing disuse atrophy. Additional study into the role that myostatin signaling plays in disuse atrophy and, in particular, muscle loss associated with the hindlimb suspension model is warranted.

In this study, we sought to further elucidate the role of the myostatin pathway in the etiology of hindlimb suspension-induced muscle loss. We utilized an anti-myostatin peptibody, a phage display-derived myostatin-neutralizing peptide fused to a murine Fc domain, and evaluated endpoint measures including hindlimb muscle masses, fiber cross-sectional area and type, and integrated hindlimb and individual muscle (soleus) strength. In addition, protein synthesis and degradation pathways were studied to establish how skeletal muscle mass was influenced by myostatin inhibition during unloading.

## Results

**Fig. 1:**
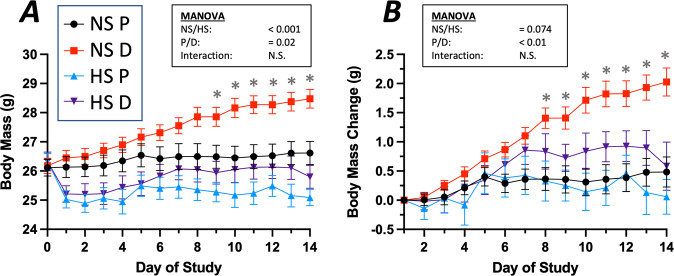
Body mass. **A** Average body mass of mice measured daily throughout the hindlimb suspension 2-week period of time. Hindlimb suspension caused a sharp drop in body mass starting 1 day after suspension compared to control mice (NS-P). Myostatin inhibition resulted in steady increases in body mass of non-suspended mice (NS-D), which became significantly greater based on Tukey’s posthoc analysis starting on Day 9. **B** Data are presented as a change in body mass relative to hindlimb suspension Day 1 to illustrate ongoing treatment effects after the initial loss of body mass due to hindlimb suspension. The repeated measures ANOVA revealed no significant effect of hindlimb suspension over this period of time but did demonstrate a significant impact of myostatin inhibition, particularly observed in the control mice (NS-D vs. NS-P). * denotes significant difference with Tukey’s posthoc analysis at *α* = 0.05. Data are presented as mean ± SEM, *n* = 18 mice/group.

Body mass and muscle masses were reduced with suspension and increased with myostatin inhibition. Body mass changes were progressive throughout the two-week suspension period (Fig. 1). Control mice (NS-P) gained approximately 0.5 g (+2.0%) in body mass over the study duration. Hindlimb-suspended mice (HS-P and HS-D) dropped significant body weight (−4.9% and −4.3%, respectively, *p* < 0.0001) after one day of suspension (Fig. 1A; the mass of HS-P mice remained unchanged through the rest of the suspension period whereas HS-D mice regained most of the Day 1 mass reduction by the end of the first week and then remained unchanged for the second week (Fig. 1B). In stark contrast, anti-myostatin peptibody-treated NS-D mice exhibited steady body mass gains throughout the study period (total 2.3 g, 8.8%), which was significantly greater than all other groups (*p* < 0.01).

In non-treated mice, hindlimb suspension (HS-P) resulted in a significant decrease in endpoint masses of quadriceps (−12.6%), calf complex (−16.4%), gastrocnemius (−12.6%), soleus (−21.6%) and TA (−11.2%) (Table 1). Neither lean body mass nor EDL mass was significantly reduced with hindlimb suspension. NS-D mice showed significant increases in whole body mass (+7.1%), lean body mass (+9.2%) and in the masses of the quadriceps (+12.3%), gastrocnemius (+11.1%), calf complex (+8.0%) and TA (+9.4%) compared to NS-P control mice. Neither soleus nor EDL endpoint masses were significantly different between non-suspended groups. Anti-myostatin peptibody treatment of hindlimb suspended mice (HS-D) partially maintained body mass, lean body mass and TA muscle masses at levels that were not significantly different from those of NS-P controls. However, the quadriceps, calf complex, gastrocnemius and soleus masses from the HS-D group were still significantly reduced (−10.9%, −11.6%, −9.6% and −24.4%, respectively) as compared to NS-P mice. HS-D mice did exhibit higher (N.S.) whole and lean body masses and masses of quadriceps, calf complex gastrocnemius, TA and EDL in relation to HS-P mice. However, only the TA was found to be significantly increased in HS-D vs. HS-P mice (*p* < 0.05).

**Table 1:** Whole body, lean body, and wet muscle masses.

					Two-way ANOVA		
	NS-P	NS-D	HS-P	HS-D	NS/HS	P/D	Int
Whole body mass (g)	26.6 ± 0.4^A^	28.5 ± 0.3^B^	25.1 ± 0.3^C^	25.8 ± 0.4^AC^	*p* < 0.001	*p* < 0.001	N.S.
Lean body mass (g)	18.4 ± 0.3^A^	20.1 ± 0.3^B^	17.6 ± 0.2^A^	18.3 ± 0.3^A^	*p* < 0.001	*p* < 0.001	N.S.
Quadriceps (mg)	222.3 ± 4.9^A^	249.6 ± 4.0^B^	194.4 ± 4.9^C^	198.0 ± 3.7^C^	*p* < 0.001	*p* < 0.001	*p* < 0.01
Calf complex (mg)	178.3 ± 2.7^A^	192.6 ± 3.7^B^	149.1 ± 3.3^C^	157.6 ± 3.1^C^	*p* < 0.001	*p* < 0.001	N.S.
Gastrocnemius (mg)	147.9 ± 3.7^A^	164.3 ± 3.4^B^	129.2 ± 3.1^C^	133.7 ± 2.9^C^	*p* < 0.001	*p* = 0.002	N.S.
Soleus (mg)	10.6 ± 0.3^A^	11.8 ± 0.5^A^	8.1 ± 0.6^B^	8.0 ± 0.4^B^	*p* < 0.001	N.S.	N.S.
TA (mg)	55.4 ± 1.0^A^	60.6 ± 0.7^B^	49.2 ± 0.8^C^	53.1 ± 0.9^A^	*p* < 0.001	*p* < 0.001	N.S.
EDL (mg)	13.1 ± 0.6^AB^	13.9 ± 0.6^A^	11.7 ± 0.4^B^	12.7 ± 0.3^AB^	*p* < 0.05	N.S.	N.S.

Data are presented as mean ± SEM. A two-way ANOVA (NS/HS; P/D) was performed with main effects indicated using *p* values. Group-wise differences were assessed using a posthoc Tukey’s test; data are significantly different when not connected by the same letter; *α* = 0.05.

**Fig. 2:**
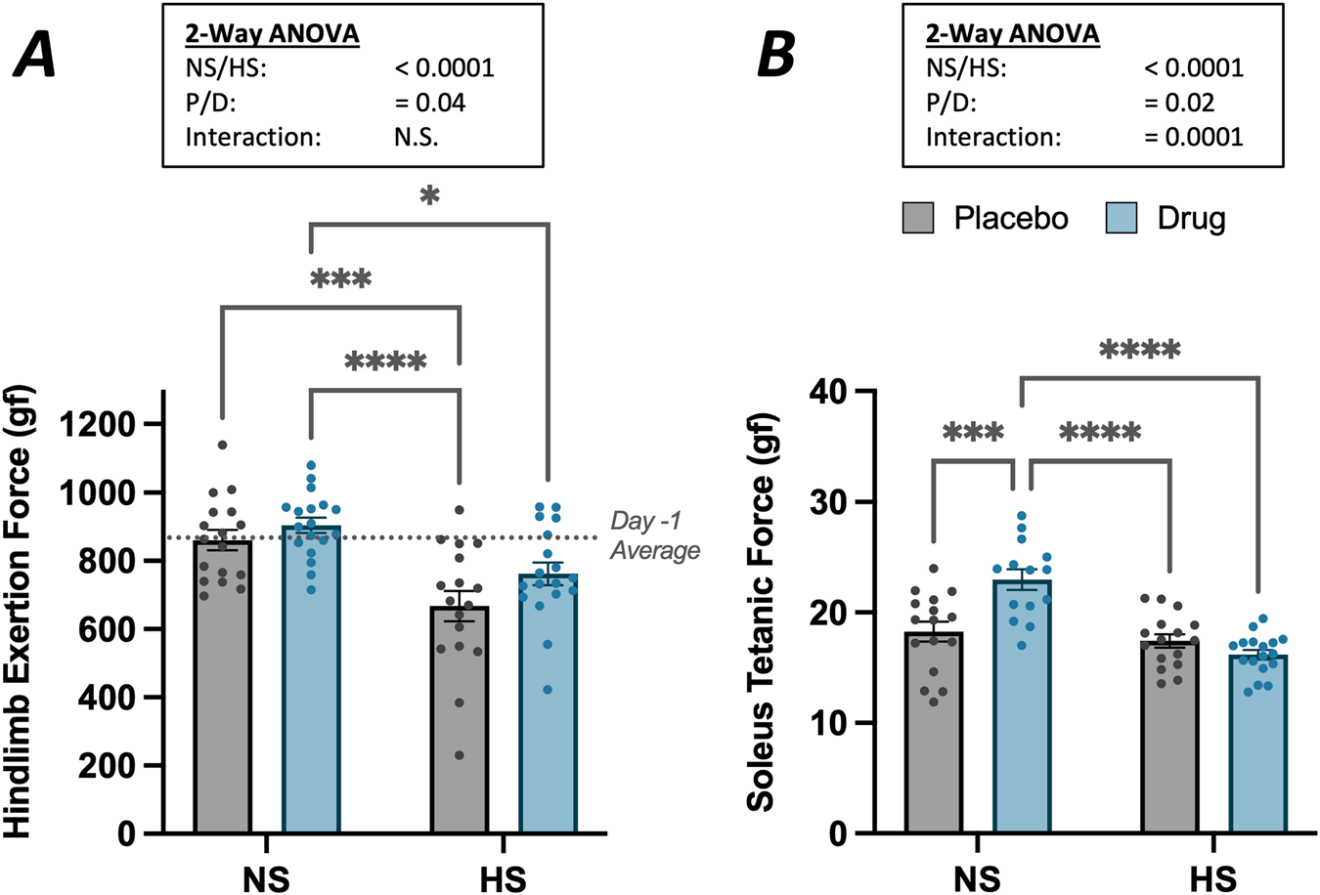
In vivo and in vitro muscle force assessment. **A** Hindlimb exertion force test (HEFT) results obtained on Day 13 of the study show significant effects of both suspension (*p* < 0.001) and treatment (*p* = 0.04) with two-way ANOVA. Tukey’s tests reveal significant differences with unloading but increases in force generation with anti-myostatin peptibody treatment did not reach significance, as indicated by dissimilar letters. Data are presented as mean ± SEM, *n* = 17–18 mice/group; *α* = 0.05. **B** Tetanic force measurement was performed in vitro from the soleus muscle. Two-way ANOVA revealed significant effects of suspension (*p* < 0.001) and treatment (*p* = 0.02) along with a significant interaction effect (*p* < 0.001). Posthoc Tukey’s revealed a significant difference with anti-myostatin peptibody treatment in non-suspended (but not in suspended) mice, as indicated by dissimilar letters. Data are reported as the mean ± SEM for each group, *n* = 15–17 mice/group; *α* = 0.05.

Myostatin inhibition partially increased in vivo strength that was diminished with hindlimb suspension. Baseline in vivo strength across all groups measured on study Day −1, one day prior to the start of treatment or suspension, was 843 ± 103 grams-force. Hindlimb force measured on day 13 of HS showed main effects of both HS (*p* < 0.001) and anti-myostatin peptibody treatment (*p* = 0.04) (Fig. 2A). Group-wise comparisons showed that in vehicle-treated mice (HS-P), suspension significantly reduced hindlimb force by −19.8% (*p* < 0.01) but that anti-myostatin peptibody did not significantly increase in vivo force production (HS-D vs. HS-P). As with individual muscle weights, there was a non-significant trend toward increased force production such that HS-D and NS-P groups were not statistically different from each other.

Soleus force production measured in vitro was partially improved with myostatin blockade. Maximum tetanic force response (P_o_) of the soleus showed significant reduction with the main effect of hindlimb suspension (*p* < 0.001) and increased with anti-myostatin peptibody treatment (*p* = 0.02). However, group-wise comparisons demonstrated that the anti-myostatin peptibody increased force production in the soleus in control (NS-D, + 22.4%) but not in unloaded mice (HS-D, Fig. 2B), a pattern similar to that of the soleus muscle masses (Table 1). Pearson’s correlation analysis showed a positive relationship (*r* = 0.55) between soleus mass and the maximum tetanic force that was produced. When taken together, this result associates mass loss in suspension with decreased tetanic force production and soleus mass gain with the myostatin inhibitor (i.e., in the unsuspended mice) with increased force production.

**Fig. 3:**
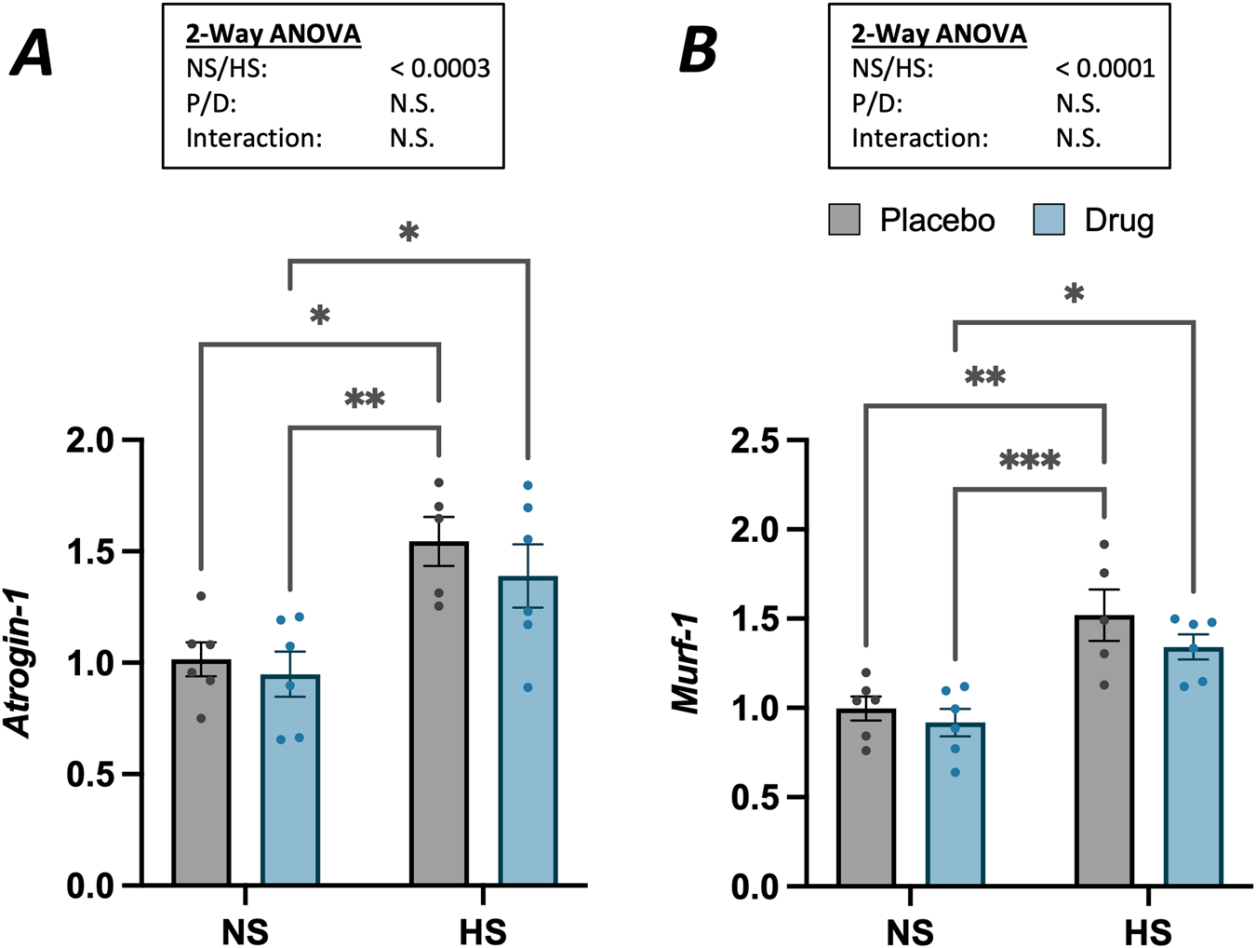
Relative gene expression levels from rt-PCR analysis of the tibialis anterior. Relative gene expression levels are shown for: **A** Atrogin-1 and **B** MuRF-1 normalized to a beta-actin control. Two-way ANOVA revealed significant increases in expression for Atrogin-1 and MuRF-1 with hindlimb suspension (*p* < 0.001) but no significant impact of myostatin inhibition. Posthoc Tukey’s comparisons showed significant differences as shown by dissimilar letters. Data are presented as mean ± SEM, *n* = 5–6 mice/group; *α* = 0.05.

Expression of key atrogenes were increased at 2 weeks of hindlimb suspension but not affected by myostatin inhibition. Atrogenes, Atrogin-1 and MuRF-1, were upregulated (*p* < 0.001) with hindlimb suspension in both the vehicle and anti-myostatin peptibody-treated groups (Fig. 3A, B, respectively), yet treatment with the inhibitor did not significantly affect Atrogin-1 or MuRF-1 expression. Based on Tukey’s pairwise comparisons, there was a trend toward decreased Atrogin-1 and MuRF-1 gene expression with anti-myostatin peptibody treatment such that HS-D and NS-P groups were not found to be statistically different from each other. At the study endpoint, IGF-1 showed no differences among the four treatment groups. We observed that Atrogin-1 and Murf-1 were negatively correlated with the TA mass (Pearson’s *r* = −0.58 and −0.50, respectively) and IGF-1 was positively correlated with TA mass (*r* = 0.45). When considered with the TA mass, which decreased with suspension and increased with drug treatment, protein degradation appeared to be a regulator of TA mass despite this muscle being subjected to chronic stretch during suspension.

**Fig. 4:**
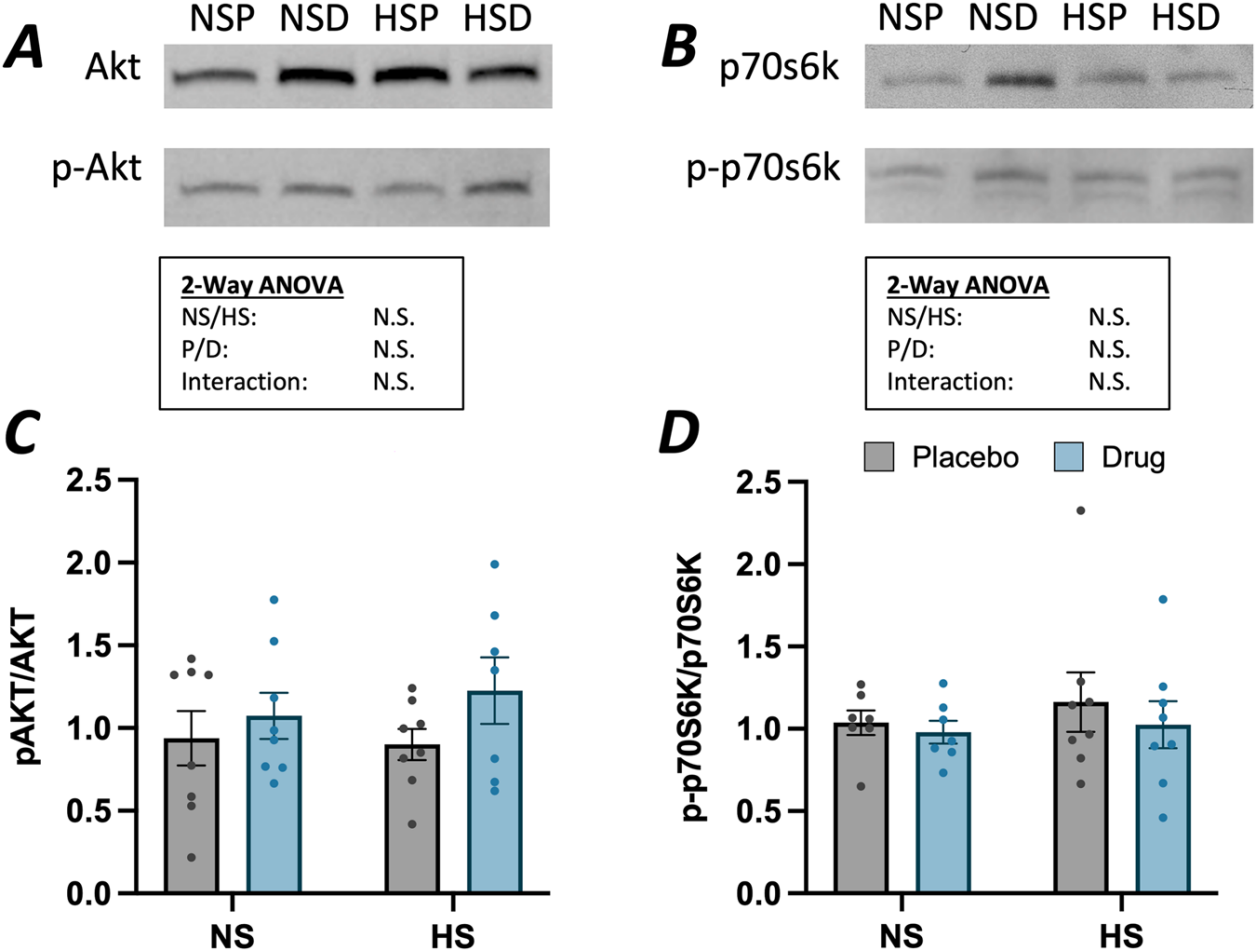
Activated levels of Akt and p70s6k protein content in the tibialis anterior. **A** Representative immunoblot of total Akt and phosphorylated Akt and **C** ratio of p-Akt to Akt levels. **B** Representative immunoblot of total p70s6k and phosphorylated p70s6k and **D** ratio of p-p70s6k to p70s6k. Two-way ANOVA revealed no effect of suspension or significant activation of pAkt/Akt. There were no effects of either suspension or drug in *p*-p70s6K/p70s6K. Posthoc Tukey’s tests revealed no differences between any groups. Data are presented as mean ± SEM, *n* = 7–9 mice/group; *α* = 0.05.

Akt activation was not increased at 2 weeks of hindlimb suspension but was elevated through inhibition of myostatin signaling. Changes in activation of Akt signaling were measured using the ratio of phosphorylated Akt (pAkt (ser 473)) to non-phosphorylated Akt (Fig. 4A; Supplemental Fig. 1A). This ratio was unaffected by two weeks of HS and administration of the myostatin inhibitor (+25.1%, N.S. increase with myostatin inhibition); as the data were variable among groups, future studies might more deeply evaluate if and how other aspects of this synthesis pathway are affected. We further assessed the potential downstream effects on activation of p70S6K (phosphorylated p70S6K/p70S6K) and found no significant differences with either HS or myostatin inhibition (Fig. 4B; Supplemental Fig. 1B). Total and phosphorylated Akt and p70s6k levels were variable and showed no differences between treatment groups. No correlations were observed between expression of proteins related to synthesis and TA mass.

**Fig. 5:**
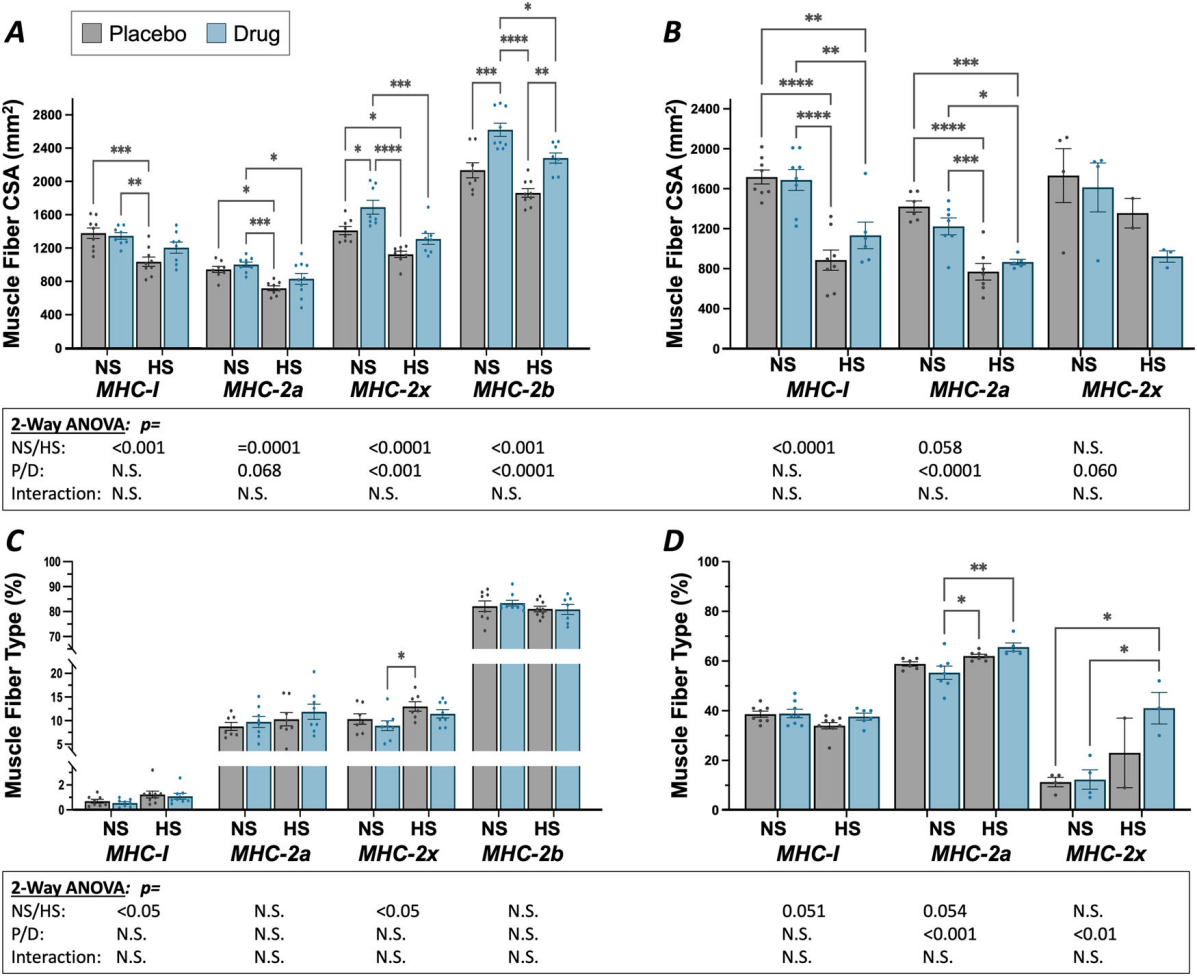
Muscle fiber cross-section area (CSA) and fiber type analyses of MHC isoform expression. **A**, **C** Gastrocnemius and **B**, **D** soleus muscles, respectively. A two-way ANOVA revealed highly significant effect of HS on CSA for both the gastrocnemius and soleus for nearly all fiber types (*p* < 0.001, exception was soleus Type IIx fibers). Posthoc Tukey’s comparisons demonstrated significant differences as indicated by dissimilar letters. Overall, group-wise comparisons in the gastrocnemius revealed a reduction in most fiber types with hindlimb suspension alone and a recovery with treatment with the anti-myostatin peptibody, most notably in Type IIb fibers. Muscle fiber CSA was significantly reduced in the soleus with unloading but showed no significant recovery with anti-myostatin peptibody treatment. Fiber type analysis revealed significant increases in fraction of fibers expressing in Type I and IIx MHC isoforms in the gastrocnemius and Type IIx MHC isoforms in the soleus. Technical difficulties prevented proper analysis of Type IIb fibers in the soleus. Group-wise comparison was done with Tukey’s posthoc analysis. Data are reported as mean ± SEM, *n* = 8–9 mice/group; *α* = 0.05.

Hindlimb suspension and myostatin inhibition caused myofiber type-specific atrophy and hypertrophy, respectively. Hindlimb suspension largely reduced muscle fiber cross-sectional area (CSA) measures in both the gastrocnemius and the soleus (Fig. 5A and C, respectively). In the gastrocnemius, hindlimb suspension of vehicle-treated mice led to significantly decreased cross-sectional area (CSA) of MHC-I (−24.8%), MHC-IIa (-23.8%) and MHC-IIx (−20.2%) stained fibers (note: MHC-IIb decreased by −12.7%; *p* = 0.056). Fiber type analysis of the gastrocnemius revealed a distribution of Type I, IIa, IIx, and IIb in the gastrocnemius of approximately 3%, 10%, 10%, and over 80%, respectively (Fig. 5B). HS resulted in a slight but significantly increased percentage of Type I (*p* = 0.014) and Type IIx (*p* = 0.016) fibers. There was no impact of HS on fiber type distribution of either Type IIa or IIb fibers in the gastrocnemius.

Soleus MHC-I and MHC-IIa fiber CSA significantly decreased with hindlimb suspension, in vehicle-treated groups (NS-P vs HS-P), by −48.4% and −45.8%, respectively. The number of MHC-IIx and MHC-IIb expressing fibers in the soleus was noticeably increased with HS (Fig. 5D); however, fluorescent staining was inconsistent and generally weak as compared to the strongly stained IIx and IIb fibers in the gastrocnemius. As a result, it proved somewhat difficult to quantify CSA or fiber type distribution especially in soleus IIb fibers.

Treatment with the anti-myostatin peptibody resulted in significant family-wise (*p* < 0.001) increases in gastrocnemius Type IIx and IIb fiber CSA but exhibited no effect on either Type I or IIa fibers (Fig. 5A). Even so, for all fiber types, myostatin inhibition resulted in a degree of normalization such that CSA was not significantly different between HS-D and NS-P control mice. Inhibition of myostatin in non-suspended mice (NS-D vs. NS-P) significantly increased gastrocnemius CSA of both Type IIx (+19.7%) and IIb (+22.7) fibers. The MHC IIb fiber CSA from HS mice also increased (+22.5%) with anti-myostatin peptibody treatment (HS-D vs. HS-P). We observed strong relationships of the gastrocnemius mass with positive correlations in Type II fibers (i.e., Pearson’s correlation coefficients with the cross-sectional area of IIA, IIB, IIX fibers were 0.53, 0.70, and 0.62, respectively). However no strong associations existed between the mass of the gastrocnemius and the percentage of any particular fiber type; there was a slight negative correlation between the gastrocnemius mass and the percentage of type I fibers, *r* = −0.25. For the soleus, myostatin inhibition had no impact on either Type I or Type IIa fiber CSA for either NS or HS mice. Myostatin inhibition also had no effect on fiber type composition for the gastrocnemius.

Tetanic force production by the soleus was moderately correlated with fiber-type expression. We observed a moderate positive correlation between soleus tetanic force production and the percentage of Type I fibers (Pearson’s *r* = 0.44) and a negative correlation with the percentage of Type IIA (Spearman’s *r* = −0.50) and IIx (Pearson’s *r* = −0.73) fibers. This finding confirms that the changes in force production by the soleus are related to fiber type shifts that occurred with suspension and myostatin inhibition.

## Discussion

In this study, we used a highly specific anti-myostatin peptibody inhibitor of myostatin to evaluate the role of myostatin signaling in hindlimb suspension muscle pathology. Myostatin is critical to the balance of protein synthesis and degradation in skeletal muscle, thus myostatin-inhibiting-therapeutics hold promise to mitigate the deleterious effects of disuse. Deletion of Mstn in hindlimb unloaded mice resulted in similar muscle atrophy as wild-type mice^47^, whereas others have reported that Mstn deletion increases muscle loss with unloading^44^. Administration of an anti-myostatin antibody attenuated skeletal muscle atrophy and functional losses in skeletal muscles from casted hindlimbs in mice^46^. Myostatin inhibition has also demonstrated therapeutic potential in more complex mouse models involving muscle wasting with disorders including muscular dystrophy, cachexia and chemotherapy, and aging^34,35,48^. This study thus sought to elucidate how myostatin is involved in disuse muscle atrophy and if myostatin inhibition might serve as a viable therapeutic option for lost muscle mass and function in spaceflight.

The mouse hindlimb suspension (HS) model mimicked many of the changes that occur with spaceflight-induced muscle atrophy and loss of function^3,43,49,50^. Two weeks of HS reduced muscle masses, fiber type, and CSA for all MHC isoforms evaluated, and measures of in vivo strength and in vitro soleus force production. Our in vivo strength assessment was noteworthy since this measurement assesses integrated strength in an awake animal that includes central nervous system activation, neuromuscular junction function, and capacity for tetanic force production in multiple muscles. Evaluation of the tibialis anterior revealed a shift towards protein degradation with unloading. MuRF-1 and MAFbx/Atrogin-1 expression was increased at study endpoint when atrogene expression has been observed to decrease to control levels^9^. Expression of these genes has been linked to greater atrophy of fast-twitch fibers in limb immobilization^51^, which we also observed. However, elevated catabolic processes were not accompanied by reduced Akt activation in contrast to other studies where Akt levels were lower at 7 and 14 days of HS^9,52^. Biomarkers of protein synthesis were not significantly different, even though p70S6K phosphorylation increased early in HS^53^ followed by reduced activation at 7 days^52^. Future work evaluating downstream targets of p70s6k would be particularly informative. Taken together, these results suggest that at our study endpoint following two weeks of HS, muscle atrophy resulted from greater protein degradation rather than reduced protein synthesis^9^.

Hindlimb suspension led to rapid body mass loss likely caused by a combination of short-term fasting and stress. Accurate measurement of food and water intake is laborious and error-prone^13^ and thus was not performed in this study. However, in a prior hindlimb suspension study of similarly aged male C57BL/6 mice we observed that body weight losses were directly associated with decreased food and water intake on the first two days of hindlimb suspension^9^. We can deduce that fasting was present in our mice at early time points, despite ad libitum access to food and water. This observation is based on the immediate drop in body mass following suspension; we also note that body mass subsequently increased starting at day 3 of suspension. Studies with unloading or denervation^9,10^ suggest two phases in response to disuse; the first marked by rapid muscle loss and large-scale gene expression changes, most notably in protein degradation, and a second phase marked by slower muscle loss along and subtle changes in gene expression. We and others have observed a rapid loss and subsequent partial recovery of body mass with HS^9,13,54^. These results during the initial phase are consistent with short-term fasting^14,55^ and increased stress/corticosterone^56,57^, which have been shown to trigger muscle atrophy independent of disuse. We observed significantly lower daily food and water consumption in the first ~2 days of HS that then returned to normal levels. Spaceflight, casting, and hindlimb suspension also increase circulating or tissue-specific levels of pro-inflammatory cytokines^13,51,58^. While not evaluated herein, inflammatory cytokines can elevate expression of the nuclear factor-kappa B (NF-κB) transcription factor, which is upstream of both myostatin and atrogenes and elevates proteolysis^59^. Muscle atrophy and associated large-scale gene expression changes during the first week of HS are likely complex and involve disuse and other extrinsic factors.

Fasting-induced muscle atrophy may occur independently of myostatin signaling. Muscle loss, including fasting^14^, psychological stress and activation of the HPA axis^56,60^, cancer cachexia^61^, sarcopenia^62^, and disuse^12,25,37,46^ are all associated with increased myostatin. Yet mice without functional myostatin still undergo fasting-induced muscle atrophy with significantly increased expression of Atrogin-1 and MuRF-1^14^. In contrast, stress-induced muscle atrophy^57^ was shown to be associated with increased myostatin, MuRF-1, and Atrogin-1 expression, and is blunted in myostatin null mice^56^. This suggests that atrophy resulting from stress and HPA axis activation is dependent on myostatin signaling. Fasting and psychological stress are also associated with disuse. We observed significantly increased MuRF-1 and Atrogin-1 expression with hindlimb suspension that occurred independently of myostatin peptibody treatment. While myostatin depletion can lead to catabolic processes in skeletal muscle, these collective observations indicate that factors other than myostatin determine muscle loss with disuse.

Inhibiting myostatin signaling resulted in only partial muscle sparing in HS mice. Myostatin inhibition in HS mice led to modest increases in muscle mass, fiber CSA (in fast fiber types), and in vivo strength. Myostatin mRNA is highest in Type IIb fibers^12^. Here, ~80% of the gastrocnemius muscle fibers expressed Type IIb, MHC thus it was not surprising that muscle mass was significantly increased in controls. However, while mass was only modestly higher, the fast-twitch fibers in the gastrocnemius underwent hypertrophy in suspended mice treated with the drug. In the TA, myostatin inhibition was associated with no measurable change in Akt activation, although we observed a decrease in the downstream factors Atrogin-1 and MuRF-1. Knockout mice for Atrogin-1 and MuRF-1 genes exhibit significant muscle sparing during hindlimb suspension^10,63^. Muscle protein degradation may be suppressed by increased levels of p-Akt and stimulate FoxO phosphorylation leading to elevated expression of Atrogin-1 and MuRF1^64^. Moreover, we have shown that atrogene expression is temporal, where Atrogin-1 and MuRF1 levels transiently, but significantly, increase in both the TA and gastrocnemius in the first week of unloading and then decline to baseline levels by 14 days^9^. Thus, it was unexpected that blocking myostatin signaling did not suppress Atrogin-1 and MuRF-1 expression at 2 weeks of HS. Notably, Carlson et al.^12^ reported that MSTN mRNA may not be required for atrophy—at least in the soleus^12^. Also, while FoxO and inflammatory factors involved in muscle degradation were not evaluated in this study, future assessments should more closely evaluate the role of this key transcription factor as well as inflammatory cytokines. An improved understanding of how myostatin interacts with pathways involved in cell mechanoresponsiveness is also needed to elucidate exactly how protein synthesis and degradation mechanisms are altered in hindlimb suspension.

In contrast to HS mice, the myostatin inhibitor had pronounced anabolic effects in normally loaded controls. Lean mass and hindlimb muscle masses increased, except for the small postural muscles. Due to the fiber-type specificity of myostatin signaling^12^, the predominantly slow fiber types in the soleus are unlikely to benefit from myostatin inhibition. However, in unsuspended mice treated with the drug, the in vitro soleus tetanic force production increased. Myostatin inhibition did not lead to any measurable increase in Akt activation, which is an upstream regulator of FoxO transcription factors^65,66^, nor did it lead to an increase in p-p70s6K/p70s6K, which indicates increased protein synthesis^31^. It is noteworthy that the expression of these proteins was measured in the TA, which showed the most robust response to myostatin inhibition. Yet in HS, this muscle is subjected to chronic stretch which is hypertrophic in immobilized limbs^67^. Evaluating a shortened muscle (e.g., the gastrocnemius) would have been informative; however, we have previously shown similar expression of atrogenes in both the TA and gastrocnemius after 14 days of HS. Thus it remains unclear if myostatin signaling and mechanisms leading to protein degradation during disuse are influenced by muscle stretch. Despite this limitation, the effects of blocking myostatin signaling in normally loaded mice follow previous research^20,29^ and demonstrate that myostatin inhibition can induce profound hypertrophy. While myostatin inhibition provided some benefit to, but lacked full recovery of, muscle mass and function in suspended mice, our results provide new insight into the role of myostatin during musculoskeletal unloading.

The lack of greater benefits of myostatin inhibition in HS mice may be due to the complex nature of the suspension protocol. Both glucocorticoids^60^ and fasting^14^ increase myostatin signaling. Glucocorticoid-induced muscle atrophy is dependent on functional myostatin^60^ while muscle atrophy during fasting occurs even in the absence of myostatin signaling^14^. Similarly, previous research shows that disuse results in increased myostatin gene expression and signaling in humans^25,36,37^ and in rodents^12,38,39^. However, similar to fasting, disuse atrophy per se occurs even in the absence of functional myostatin signaling^44^. Also, muscle stretch reduces myostatin expression and increases activation of protein synthesis pathways^68,69^ and partially counteracts muscle atrophy with unloading^70^. Thus, research utilizing the hindlimb suspension model should be viewed in light of the various physiological and physical stimuli present along with HPA activation, increased circulating corticosterone, fasting, and biomechanics.

Processes that occur in addition to ubiquitin-proteasome-dependent proteolysis may have contributed to our results. For example, spaceflight in astronauts^71^ and hindlimb unloading in adult mice^11^ cause autophagy of skeletal muscle cells. Like the ubiquitin-proteosome pathway, autophagy is also under FoxO regulation and, when activated, promotes muscle degeneration. However, autophagy flux is required to preserve muscle mass and maintain myofibers^72^, which is postulated to occur via stimulation of mTor kinase activity and protein synthesis^11^. Additional studies are needed to fully probe the mechanisms underlying muscle loss using the hindlimb unloading model.

Inhibiting additional ligands that signal through the primary myostatin receptor may protect against muscle loss. Myostatin is known to signal through transmembrane activin Type IIB receptors (ActRIIB) along with Activin A and other ActRIIB ligands^28,34^. Increased levels of ActRIIB in fast muscle fiber types may partly explain the predominant impacts of HS in fast versus slow muscle fibers^73^. Inhibiting this signaling pathway with a soluble decoy ActRIIB receptor induces hypertrophy in a fiber-type independent manner^74^ and prevents limb immobilization muscle atrophy, but not denervation atrophy^75^. Our use of a decoy receptor, which inhibits myostatin along with Activin A and other ligands, appears to more fully prevent muscle atrophy, at least in the TA, then inhibiting myostatin alone, as in our study. Signaling with Activin A or other TGF-β family members may thus play an important role in HS-induced muscle atrophy, where the use of a therapeutic to block ActRIIB receptor signaling may ultimately demonstrate efficacy.

Myostatin and associated signaling pathways increase during exposure to microgravity during spaceflight in mice^41,42^; thus, myostatin is a potential target to prevent spaceflight-induced muscle loss. Lee et al. showed increased myostatin signaling and significant skeletal muscle mass losses in mice flown on the ISS but muscle sparing and growth in mice treated with a decoy ActRIIB receptor^42^. Smith et al. demonstrated that a monoclonal antibody to myostatin significantly increased mouse skeletal muscle masses following 6-weeks on the ISS^43^. Interestingly, gene expression of myostatin or MuRF-1 was not increased at our study endpoint in untreated mice suggesting that muscle loss plateaus after two weeks of unloading. In addition, unlike hindlimb suspension, muscle loss in MuRF-1 knockout mice is not prevented during spaceflight^49^. Taken together, these findings suggest that spaceflight may cause muscle atrophy through mechanisms different from that of HS.

In summary, blocking myostatin signaling using an anti-myostatin-specific peptibody only partially mitigated muscle atrophy and functional strength following 14 days of hindlimb suspension of male mice. Additional direct comparisons between spaceflight and hindlimb suspension will be required to fully assess the utility of the suspension model to address spaceflight musculoskeletal problems. Results from this study also suggest that there could be therapeutic potential for myostatin inhibition in disuse muscle wasting and perhaps even in astronauts assigned to long-duration space missions. Further work is warranted to ascertain the full potential of blocking this important pathway in musculoskeletal unloading and in spaceflight.

## Methods

### Anti-myostatin peptibody

A murine version of myostatin-inhibiting peptibody was provided by Amgen Inc. (Thousand Oaks, CA). Structurally, the anti-myostatin peptibody utilized was a phage display-derived myostatin-neutralizing peptide fused to an Fc domain of murine IgG1. In cell-based Smad2/3 signaling assays, the anti-myostatin peptibody exhibited potent myostatin-neutralizing activity (IC_50_ < 1 nM against 4 nM myostatin).

### Animals

Seventy-two male, 12-week-old C57BL/6J mice (Jackson Labs, Bar Harbor, ME) were divided into four groups (*n* = 18/group): non-suspended and hindlimb-suspended treated with placebo (saline, s.c.) (NS-P and HS-P, respectively) or an anti-myostatin peptibody (NS-D and HS-D, 10 mg/kg, s.c.). Dosage and frequency of peptibody administration were determined from separate pharmacokinetic studies in mice performed at Amgen Inc. (unpublished data). Hindlimb-suspended mice were positioned at a 30° head down angle for 14 days as previously described^9,54^. Injections of the myostatin inhibitor were given on days 0, 3, 6, 9, and 12 of the study. Mice were housed individually with a 12-h light/dark cycle and provided ad libitum access to food and water, and monitored daily for body weight and health status. All experimental protocols involving live mice were approved by the University of Colorado Institutional Animal Care and Use Committee.

### Hindlimb exertion force test

An in vivo functional assay to quantify the force produced predominately by the hindlimb muscles, the “Hindlimb Exertion Force Test” (HEFT), was performed as previously described^76^. Briefly, HEFT evaluates dominant hindlimb muscles used during an evoked escape response (electrical stimulus, 1.5 mA, 0.3 s administered to the tail) and was designed as an alternative to the more conventional grip strength measurement. Two stimuli are applied to the tail (with a 2 min rest) which is affixed to a force transducer; the force responses were recorded through a custom developed Labview™ v7.1 (National Instruments Corporation, Austin, TX) code. Mice were subjected to HEFT on study days −1 and 13.

### In vitro soleus force measurements

Electrical stimulation of the soleus muscle was performed to measure strength, fatigue, and recovery from fatigue following all treatments. A detailed description of soleus electrophysiology experiments is described in Hanson et al.^9^. Prior to sacrifice, mice were deeply anesthetized with sodium pentobarbital (90 mg/kg) and chlorohydrate (120 mg/kg). The soleus was surgically removed and placed in a modified Kreb’s solution with pH 7.4, at 37 °C, gassed with 95% O_2_/5% CO_2_, and circulated at 10 mL/min. Muscles, spanned by parallel platinum plate electrodes, were mounted horizontally using #5–0 silk sutures with one hook attached to an isometric Harvard-Hugo Sachs F30 force transducer (Harvard Apparatus, Holliston, MA). Muscles were adjusted to optimal resting length (L_o_) to obtain maximum twitch force. Stimulation amplitude, at the supramaximal threshold level of 80 mA, consisted of either a single pulse (twitch response, 0.2 ms) or defined pulse trains (tetanus response, P_o_; 0.2 ms pulse width, 140 Hz, 500 ms). Data acquisition and stimulus initiation were controlled with a custom LabVIEW™ v7.1 (National Instruments Corporation, Austin, TX) program.

### Tissue collection

At sacrifice, lean body mass (LBM) was recorded as the carcass mass after removal of head, skin, and subcutaneous adipose tissue. Hindlimb muscles including the left side quadriceps, tibialis anterior (TA), extensor digitorum longus (EDL), soleus, plantaris were collected, weighed and snap frozen in liquid nitrogen for subsequent analysis. The calf complex (soleus/gastrocnemius/plantaris) from the right limb was collected, frozen on cork in liquid nitrogen cooled isopentane and stored at −80 °C for subsequent histological analysis.

### Muscle fiber CSA and MHC quantification

Immunohistochemical analysis was performed on the calf complex to quantify cross-sectional area (CSA) of individual fibers and expression of myosin heavy chain (MHC) isoforms I, IIa, IIx, and IIb. Serial sections from the calf complex (10 µm) were made using a Cryostat Microtome (Tissue Tek II, Sakura Finetek USA, Inc., Torrance, CA), fixed to gelatin-coated glass slides, and stored at −20 °C. Immunostaining was used to determine the myosin heavy chain (MHC) fiber type in each section of muscle as previously described^3,9^. Briefly, muscle sections incubated in a permeabilizing/blocking solution (P/BS: 0.12% bovine serum albumin (BSA), 0.12% non-fat dry milk (NFDM), 0.01% Triton-X) containing 0.05% normal goat serum and 0.02% anti-laminin antibody; rinsed 3× in PBS; and placed in a primary antibody solution (1 h, room temperature or overnight at 4 °C). The primary antibodies used to visualize MHCs included: MHCslow (Novacastra, Newcastle upon Tyne, UK), for MHC-I; SC-71 for MHC-IIa; 6H1 for MHC-IIx; and BF-F3 for MHC-IIb. Following primary antibody incubation, sections were rinsed and incubated in PBS, and were then probed (1 h, room temperature) with secondary antibodies (Jackson ImmunoResearch Laboratories, West Grove, PA): FITC- or Texas Red-conjugated goat anti-mouse IgG (NCSslow or SC-71) or IgM (BF-F3 or 6H1), and goat anti-rabbit FITC (laminin). Sections were mounted using VectraShield (Vector Labs, Burlingame, CA) and stored at 4 °C for further analysis.

Muscle sections were imaged at 4–20× (Zeiss Axiskop40FL, Carl Zeiss, Inc., Thornwood, NY) and evaluated for CSA and determine fiber type percentage (NIH Image J software, NIH, Bethesda, MD). At minimum, 50 fibers were measured from three to five non-overlapping, all-inclusive regions of the muscle section to determine CSA, and 500–1000 fibers within medial and distal regions were counted to determine the percentage of fibers expressing each MHC. Where distinct regional differences of fiber type occurred, the counts were weighted to the relative area of measurement.

### Protein isolation and quantification

Tibialis anterior (TA) lysates were prepared by homogenization in a complete lysis buffer (PBS, 0.5% Triton-X, and 1 mM EDTA, 1 tablet of protease inhibitor (Roche Diagnostics Corp., Indiana, IN), 1 mM Phenlymethylsulfonyl Fluoride, 1 mM Glycerol-2-phosphate, 1 mM Sodium Molybdate, 1 mM Sodium Pyrophosphate, 1 mM Sodium Vanadate, and 1 mM Sodium Fluoride). The protein content of the soluble fraction was determined using the Lowry assay and analyzed in a spectrometer at 750 nm for absorbance. Western blot analysis was performed on TA muscle samples and blots were probed for levels of Akt, phospho-Akt (ser473), p70s6k, and phospho-p70s6k proteins. Serial dilutions of protein lysates were loaded on SDS-PAGE gels to determine appropriate loading concentrations. A laemmli/β-mercaptoethanol (20:1) buffer (Bio-Rad, Hercules, CA) was added 1:1 with the lysates and proteins denatured by boiling in water for 5 min. Samples were cooled and loaded into an appropriate resolving SDS-PAGE gel (7.5% Tris-HCl) and run at 200 V for 30–35 minutes, or until protein bands reached the bottom of the gel. Each transfer was performed onto polyvinylidene fluoride (PVDF) filters at 100 V for 1 h and continuously stirred. The PVDF membrane was rinsed twice for 5-min each in TBS-T and blocked for 1 h at room temperature (TBS, 0.1% Tween-20, 5% NFDM) before being incubated in primary antibody at 4 °C overnight (1000:1 dilution of stock antibody in TBS-T with 5% BSA; all antibodies purchased from Cell Signaling Technology). The filter was then rinsed in TBS-T (3 × 5 min), incubated in a secondary antibody incubated for 1 h at room temperature with secondary antibody (2,000:1 dilution of horseradish peroxidase (HRP)-tagged goat anti-rabbit IgG in TBS-T with 5% nonfat milk), and rinsed a final time in TBS-T (three time for 5-min). Each membrane was stained with Ponceau S to ensure equal loading per lane. For visualization of individual protein bands following antibody incubations, membranes were incubated in Western Lightning Reagent (PerkinElmer, Boston, MA) chemiluminescent solution for 1 min and exposed to X-OMAT autoradiographic film (Kodak, New Haven, CT). Protein bands were quantified with NIH ImageJ software through densitometric analysis. All blots derived from the same experiment and were processed in parallel.

### RNA isolation and rt-PCR analysis

Total RNA was isolated from the tibialis anterior muscle by homogenizing each frozen muscle in 1 mL Trizol Reagent (Molecular Research Center, Inc., Cincinnati, OH) according to the manufacturer’s instructions. First-strand cDNA synthesis was performed on 2 μg of total RNA using Superscript II and random primers (Invitrogen Corp., Carlsbad, CA) according to the manufacturer’s instructions. Quantitative, real-time PCR was performed using the absolute quantitation, standard curve method on an Applied Biosystems 7500 thermocycler with either Taqman Master Mix (both from Applied Biosciences, Foster City, CA), gene-specific primers (12.5 μM), and 4 ng of cDNA per reaction well (3 replicate wells per sample). All reactions were run in duplicate with both the primer/probe sets for Igf-1, Atrogin-1, or MuRF-1 and the primer/probe sets for β-actin or 18 s within the same reaction to check for compatibility. Each unknown sample was run in triplicate to correct for variances in loading. All comparison groups were run on the same plate to minimize plate-to-plate variations. A standard curve ranging from 25 to 0.01 µg of mouse TA cDNA was run in duplicate on each plate to produce a standard curve for quantification. The Applied Biosystems 7500 Software v2.0.2 was used to visualize the data and calculate the mean quantities of each triplicate sample. All values are expressed as the mean of the triplicate measure for the unknown sample (Igf-1, Atrogin-1, or MuRF-1) divided by the mean of the triplicate measure of β-actin for each sample. The Taqman probe sets for Igf-1, Atrogin-1, and MuRF-1 were purchased from Applied Biosystems (Foster City, CA). The gene-specific primers were: Igf1 sense 5′-CACTCATCCACAATgCCTgTCT-3′, Igf1 antisense 5′-CTgAgCTggTggATgCTCTTC-3′; Atrogin-1 sense 5′-CTTTCAACAgACTggACTTCTCgA-3′, Atrogin-1 antisense 5′-CAgCTCCAACAgCCTTACTACgT-3′; Murf1 sense 5′-gTgAggTTgCCCCTTTgCAA-3′, Murf1 antisense 5′-TggAgATgCAgTTACTCAgC-3′; 18s sense 5′-gCCgCTAgAggTgAAATTCTT-3′, 18s antisense 5′-CTTTCgCTCTggTCCgTCTT-3′; and β-actin sense 5’-GTGACGTTGACATCCGTAAAGA-3’, β-actin antisense 5’-GCCGGACTCATCGTACTCC-3’.

### Statistical analysis

Data are presented as mean ± SEM. Body weights were evaluated with MANOVA; groups were compared using Tukey’s posthoc analysis. Statistical analyses comparing family-wise factors of unsuspended vs. hindlimb suspended and placebo vs. drug were performed using a two-way ANOVA with posthoc Tukey’s test. Statistical significance is reported at the *P* < 0.05 level for all analyses, unless otherwise indicated, and *p*-values are reported for *p* < 0.10. Data were assessed for normality using the Shapiro-Wilk test (*α* = 0.05). Pearson’s correlation was used to evaluate for relationships between two independent factors where data passed normality tests; when data were not normal, a nonparametric Spearman’s correlation analysis was performed.

### Reporting summary

Further information on research design is available in the Nature Research Reporting Summary linked to this article.

## Supplementary information

Supplement

npj Reporting Summary

## Data Availability

The datasets generated during and/or analyzed during the current study are available from the authors on reasonable request. Please contact the corresponding author for further information related to the work in this manuscript.
