# Supplementary material for: Inhibiting myostatin signaling partially mitigates structural and functional adaptations to hindlimb suspension in mice
Source: NPJ Microgravity. 2023 Jan 16;9:2. doi: 10.1038/s41526-022-00233-4 (PMC9842652; doi:10.1038/s41526-022-00233-4)
Supplement: Supplementary file 1 — Supplement [file 41526_2022_233_MOESM1_ESM.docx]

**Inhibiting Myostatin Signaling Partially Mitigates Structural and Functional Adaptations to Hindlimb Suspension in Mice**

Andrea M. Hanson, Mary H. Young, Brooke C. Harrison, Xiaolan Zhou, HQ Han, Louis S Stodieck, Virginia L. Ferguson

**Supplemental Data**


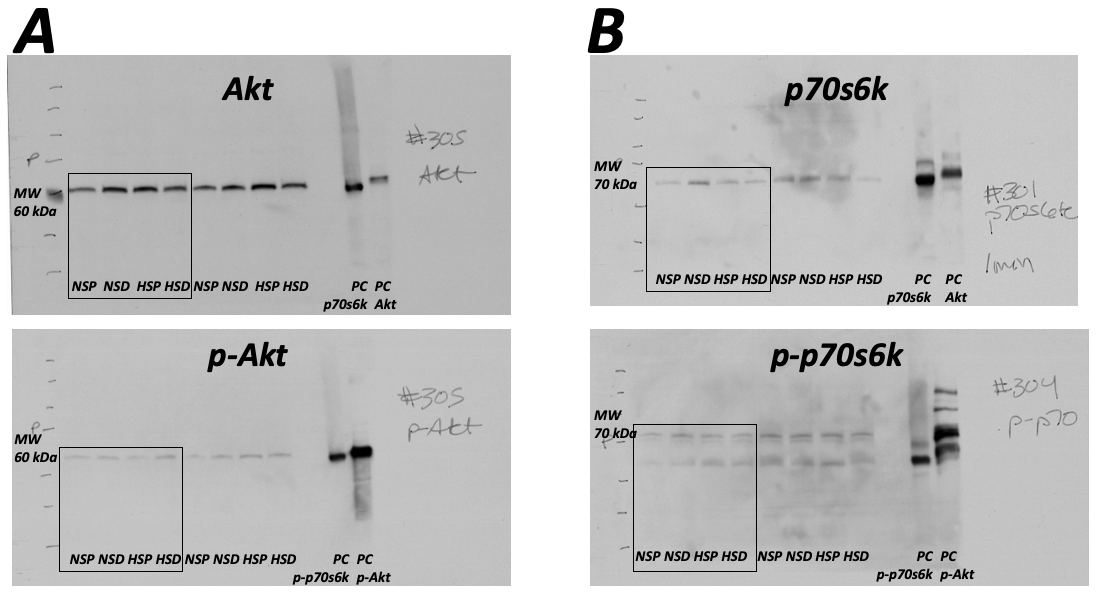


**Supplementary Figure 1. Unprocessed scans of blots used to evaluate for Akt, phosphorylated Akt (p-Akt), p70s6k, and phosphorylated p70s6k (p-p70s6k) protein content in the tibialis anterior.** Samples from eight different animals were evaluated in lanes 1-8 of each blot, with groups labeled as follows: non-suspended placebo (NSP), non-suspended drug (NSD), hindlimb suspended placebo (HSP), and hindlimb suspended drug (HSD). The protein marker covering the expected molecular weight range was loaded to the wells labeled by each protein name and “PC” for “Protein Control” (e.g., “PC-Akt”) to accurately estimate the detected proteins in lanes 9 and 10. Molecular weight (MW) is shown for each protein assayed. All blots derived from the same experiment and were processed in parallel.

Note: Boxes overlaid on each blot indicate lanes shown in Figure 4, panels A and B.
