# Supplementary material for: Inhibiting myostatin signaling partially mitigates structural and functional adaptations to hindlimb suspension in mice
Source: NPJ Microgravity. 2023 Jan 16;9:2. doi: 10.1038/s41526-022-00233-4 (PMC9842652; doi:10.1038/s41526-022-00233-4)
Supplement: Supplementary file 2 — npj Reporting Summary [file 41526_2022_233_MOESM2_ESM.pdf]

nature portfolio

Corresponding author(s): Virginia L. Ferguson

Last updated by author(s): Sep 15, 2022

Reporting Summary

Nature Portfolio wishes to improve the reproducibility of the work that we publish. This form provides structure for consistency and transparency in reporting. For further information on Nature Portfolio policies, see our Editorial Policies and the Editorial Policy Checklist.

Statistics

For all statistical analyses, confirm that the following items are present in the figure legend, table legend, main text, or Methods section.

n/a	Confirmed	
☐	☒	The exact sample size (*n*) for each experimental group/condition, given as a discrete number and unit of measurement
☐	☒	A statement on whether measurements were taken from distinct samples or whether the same sample was measured repeatedly
☐	☒	The statistical test(s) used AND whether they are one- or two-sided<br>*Only common tests should be described solely by name; describe more complex techniques in the Methods section.*
☐	☒	A description of all covariates tested
☐	☒	A description of any assumptions or corrections, such as tests of normality and adjustment for multiple comparisons
☐	☒	A full description of the statistical parameters including central tendency (e.g. means) or other basic estimates (e.g. regression coefficient) AND variation (e.g. standard deviation) or associated estimates of uncertainty (e.g. confidence intervals)
☐	☒	For null hypothesis testing, the test statistic (e.g. *F*, *t*, *r*) with confidence intervals, effect sizes, degrees of freedom and *P* value noted<br>*Give P values as exact values whenever suitable.*
☒	☐	For Bayesian analysis, information on the choice of priors and Markov chain Monte Carlo settings
☐	☒	For hierarchical and complex designs, identification of the appropriate level for tests and full reporting of outcomes
☐	☒	Estimates of effect sizes (e.g. Cohen's *d*, Pearson's *r*), indicating how they were calculated

Our web collection on statistics for biologists contains articles on many of the points above.

Software and code

Policy information about availability of computer code

Data collection	no code was generated in support of this work.
Data analysis	Software used for statistical analysis included JMP Pro 16, Prism 9 was used for statistics and graphing. LabVIEW™ v7.1 was used for data acquisition for muscle strength testing. NIH Image J was used for analysis of Western blots and immunohistochemical staining. Applied Biosystems 7500 Software v2.0.2 was used for quantitative PCR.

For manuscripts utilizing custom algorithms or software that are central to the research but not yet described in published literature, software must be made available to editors and reviewers. We strongly encourage code deposition in a community repository (e.g. GitHub). See the Nature Portfolio guidelines for submitting code & software for further information.

Data

Policy information about availability of data

All manuscripts must include a data availability statement. This statement should provide the following information, where applicable:

- Accession codes, unique identifiers, or web links for publicly available datasets
- A description of any restrictions on data availability
- For clinical datasets or third party data, please ensure that the statement adheres to our policy

The datasets generated during and/or analyzed during the current study are available from the authors on reasonable request.

Human research participants

Policy information about studies involving human research participants and Sex and Gender in Research.

Reporting on sex and gender	N/A
Population characteristics	N/A
Recruitment	N/A
Ethics oversight	N/A

Note that full information on the approval of the study protocol must also be provided in the manuscript.

Field-specific reporting

Please select the one below that is the best fit for your research. If you are not sure, read the appropriate sections before making your selection.

☒ Life sciences ☐ Behavioural & social sciences ☐ Ecological, evolutionary & environmental sciences

For a reference copy of the document with all sections, see nature.com/documents/nr-reporting-summary-flat.pdf

Life sciences study design

All studies must disclose on these points even when the disclosure is negative.

Sample size	This study employed a 2x2 factorial design. Numbers of samples for specific analyses, as well as total animal numbers, were calculated using a power analysis from two prior, but similar (unpublished), studies in our lab using similar (but less effective) myostatin inhibitor therapeutics. The total sample size of n = 18 mice / group was chosen to enable simultaneous analysis of the same tissues. Number of samples for each assay were included in the figure captions, and data points are shown on each plot wherever possible (excepting figure 1, where showing all data points makes the figure not readable).
Data exclusions	No data exclusions were pre-established. Data were only excluded for any assay where there was a damaged sample (e.g., damaged during tissue harvesting) or other technical difficulty (e.g., analysis of Type IIb fibers in the soleus - this is noted in the Figure 5 caption where these data are presented).
Replication	For Western blotting and immunohistochemical staining of skeletal muscle fiber type, we used antibodies that have been previously authenticated by ours and other labs. Our data were also evaluated using appropriate statistical methods, and were evaluated in multiple ways (ranging in conservativeness) to ensure that we reported only results in which we are confident are robust. Also note that we report p values up to 0.10 rather than applying an arbitrary cut off at 0.05 to allow for readers to interpret significance, rather than telling readers what data are/are not statistically different. We generated plots showing all data points where possible (except for figure 1, where showing all data points made the plot unreadable).
Randomization	Mice for this study were selected from a larger population of mice, where only the middle two quartiles were used (i.e., the lightest and heaviest mice were not used in this study). Mice were randomly assigned to treatment groups. Tissues were assigned a randomly generated 6-digit number (for de-identification) and a separate 3 digit number that correlated to treatment group; the 6-digit number was used throughout all data analyses where subjectivity could potentially bias the measured outcomes (e.g., MHC fiber typing). Samples were analyzed in an order that randomized treatment groups.
Blinding	Using a randomly generated 6 digit number that we used to effectively deidentify each mouse, operators were blinded to the treatment group in which each sample originated. Only after collection of all data were the samples re-identified for statistical analysis.

Reporting for specific materials, systems and methods

We require information from authors about some types of materials, experimental systems and methods used in many studies. Here, indicate whether each material, system or method listed is relevant to your study. If you are not sure if a list item applies to your research, read the appropriate section before selecting a response.

Materials & experimental systems

n/a	Involved in the study	
☐	☒	Antibodies
☒	☐	Eukaryotic cell lines
☒	☐	Palaeontology and archaeology
☐	☒	Animals and other organisms
☒	☐	Clinical data
☒	☐	Dual use research of concern

Methods

n/a	Involved in the study	
☒	☐	ChIP-seq
☒	☐	Flow cytometry
☒	☐	MRI-based neuroimaging

Antibodies

Antibodies used

Primary antibodies for IHC: MHCslow (Novacastra, Newcastle upon Tyne, UK), for MHC-I; SC-71 for MHC-IIa; 6H1 for MHC-IIx; and BF-F3 for MHC-IIb; and secondary antibodies (Jackson ImmunoResearch Laboratories, West Grove, PA): FITC- or Texas Red-conjugated goat anti-mouse IgG (NCSslow or SC-71) or IgM (BF-F3 or 6H1), and goat anti-rabbit FITC (laminin).

For Western blotting: antibodies for Akt, phospho-Akt (ser473), p70s6k, and phospho-p70s6k purchased from Cell Signaling Technology

Validation

Novacastra validation statement: "Each antibody in the range has been independently evaluated* by external QA, NordiQC, in comparison with equivalent products from other vendors. "

Jackson Immunoresearch statement: "Whole IgG antibodies are isolated as intact molecules from antisera by immunoaffinity chromatography. They have an Fc portion and two antigen binding Fab portions joined together by disulfide bonds and therefore they are divalent. The average molecular weight is reported to be about 160 kDa."

Cell Signaling Technology statement: "To ensure our antibodies will work in your experiment, we adhere to the Hallmarks of Antibody Validation™, six complementary strategies that can be used to determine the functionality, specificity, and sensitivity of an antibody in any given assay. CST adapted the work by Uhlen, et. al., (“A Proposal for Validation of Antibodies.” Nature Methods (2016)) to build the Hallmarks of Antibody Validation, based on our decades of experience as an antibody manufacturer and our dedication to reproducible science."

Animals and other research organisms

Policy information about studies involving animals; ARRIVE guidelines recommended for reporting animal research, and Sex and Gender in Research

Laboratory animals

male, 12 week-old C57BL/6 mice

Wild animals

N/A

Reporting on sex

Only male mice were used. Sex was not considered as a biological variable to reduce animal numbers. Had we included both males and females in this study, animal numbers would have had to be doubled which was not scientifically justified.

Field-collected samples

N/A

Ethics oversight

University of Colorado at Boulder IACUC approved a protocol for the treatment of mice in this study (this information is also provided in the manuscript)

Note that full information on the approval of the study protocol must also be provided in the manuscript.
